# The triglyceride glucose index as a biomarker for different glucose disorders in overweight and obese children and adolescents

**DOI:** 10.1007/s00431-025-06632-5

**Published:** 2025-12-05

**Authors:** Ola Taha, Lobna Abdallah, Adel Erfan, Radwa Elsharaby, Doaa El Amrousy

**Affiliations:** 1 Pediatric Department, Faculty of Medicine, Tanta University, Tanta, Egypt; 2 Ministry of Health and Population, Tanta, Egypt; 3 Clinical Pathology Department, Faculty of Medicine, Tanta University, Tanta, Egypt

**Keywords:** TyG index, Insulin resistance, Impaired glucose tolerance, Impaired fasting glucose, Type 2 DM, Obesity, Childhood

## Abstract

The triglyceride/glucose index (TyG index), a formula derived from fasting triglyceride (TG) and glucose levels, has emerged as a simple and reliable alternative marker for estimating insulin resistance (IR). This study aimed to evaluate the potential utility of the TyG index in predicting different glucose disorders (GD) in obese and overweight children. This prospective study included 60 children aged 5 to 15 years with overweight or obesity. They were divided into two equal groups: Group 1 consisted of children with obesity (BMI > 95th percentile), and Group 2 comprised overweight children (BMI 85th—95th percentile). The control group consisted of 30 healthy children with normal BMI, matched for age and sex. TG, fasting blood glucose (FBG), oral glucose tolerance test, 2-h postprandial glucose, the TyG index, homeostatic model assessment for IR (HOMA-IR), and glycated hemoglobin (HbA1c) were measured in all participating children. TG, HbA1c, FBG, and the TyG index were significantly elevated in both groups 1 and 2 compared with the control group. There was a strong positive correlation between TyG index and (HbA1C, FBG, HOMA IR, and 2-h postprandial glucose). The TyG index demonstrated a strong predictive ability for type 2 diabetes, impaired glucose tolerance, impaired fasting glucose, and IR with cut-off values of 4.57, 4.52, 4.43, and 4.34, respectively. These cut-off values were associated with sensitivities of 100%, 100%, 88.9%, and 71.4%, and specificities of 95.3%, 95.1%, 88.3%, and 100%, respectively.

*Conclusion*: The TyG index is a promising and reliable marker for detecting different glucose disorders in obese and overweight children.

**Table:** 

**What is Known:** • *The triglyceride/glucose index (TyG index), a formula derived from fasting triglyceride (TG) and glucose levels, has emerged as a simple and reliable alternative marker for estimating insulin resistance (IR) in pediatrics and adults*.	
**What is New:** • *We investigated, for the first time, the potential utility of the TyG index in predicting different glucose disorders (GD) such as type 2 diabetes mellitus (T2DM), impaired glucose tolerance, impaired fasting glucose as well as IR in obese and overweight children*. • *We found that the TyG index is a promising and reliable marker for detecting different glucose disorders in obese and overweight children*.

**What is Known:**

• *The triglyceride/glucose index (TyG index), a formula derived from fasting triglyceride (TG) and glucose levels, has emerged as a simple and reliable alternative marker for estimating insulin resistance (IR) in pediatrics and adults*.

**What is New:**

• *We investigated, for the first time, the potential utility of the TyG index in predicting different glucose disorders (GD) such as type 2 diabetes mellitus (T2DM), impaired glucose tolerance, impaired fasting glucose as well as IR in obese and overweight children*.

• *We found that the TyG index is a promising and reliable marker for detecting different glucose disorders in obese and overweight children*.

## Introduction

Childhood obesity has become one of the most significant public health issues worldwide. The rising rates of childhood obesity have resulted in multiple serious obesity-related health problems that not only endanger those affected but also place a significant strain on the healthcare system [1].

Childhood obesity is linked to comorbidities that affect nearly every system in the body, including, but not limited to, the endocrine, gastrointestinal, pulmonary, cardiovascular, and musculoskeletal systems. Many of the comorbidities seen in youth with obesity, such as insulin resistance (IR), prediabetes, and type 2 diabetes mellitus (T2DM), were once thought to be “adult” diseases. The severity of these comorbidities generally increases with the degree of obesity [2].

In obesity, adipocytes begin to release significant amounts of adipokines and proinflammatory cytokines that trigger obesity-related inflammation and further promote IR progression. IR with compensatory hyperinsulinemia is believed to be the earliest impairment that precedes subsequent changes in insulin secretion, ultimately leading to chronic hyperglycemia and clinically diagnosed diabetes [3]. Obesity-related genetic variants have been linked to T2DM risk through increased IR and heightened β-cell function as a compensatory mechanism against IR [4]. Triglycerides also contribute to insulin resistance. Hepatic triglyceride content is a strong predictor of hepatic insulin resistance, and intramyocellular triglycerides are associated with muscle insulin resistance [5].

The triglyceride/glucose index (TyG index), a formula derived from fasting triglyceride (TG) and glucose, has been used as an alternative tool to estimate IR compared to the hyperinsulinemic-euglycemic clamp and Homeostatic Model Assessment for Insulin Resistance (HOMA-IR). The TyG index is calculated using the following formula: Ln [fasting triglycerides (mg/dL) × fasting glucose (mg/dl)]/2. Additionally, it has demonstrated higher prediction capability than other traditional markers of IR, such as triglycerides/high-density lipoprotein cholesterol (TG/HDL-c) [6].

The TyG index has been widely recognized as a simple, cost-effective, and reliable surrogate marker of insulin resistance, particularly in adult populations [7]. Recent studies have also explored its applicability in children and adolescents, demonstrating significant associations with insulin resistance and cardiometabolic risk factors [6–8]. However, data regarding the diagnostic utility of the TyG index for identifying specific glucose metabolism disorders—such as IR, prediabetes, and T2DM in pediatric populations remain limited. Therefore, the primary aim of the present study was to evaluate the potential utility of the TyG index in predicting these glucose disorders (GD) in obese and overweight children.

## Methods

### Study design and setting

This prospective cross-sectional study was conducted on 60 obese and overweight children attending the Endocrinology Unit of the Pediatric Department, Faculty of Medicine, Tanta University, from March 2022 to June 2024.

### Compliance with ethical standards

The study was approved by the local Ethics Committee of the Faculty of Medicine at our University with approval code: 35190/1/22. It was conducted in accordance with the ethical standards outlined in the 1964 Declaration of Helsinki and its later amendments. The children were enrolled after obtaining informed consent from their parents or legal guardians.

The included children were divided equally into two groups:

- Group 1: included 30 children with obesity (body mass index [BMI] ≥ 95th centile).
- Group 2: included 30 children with overweight (BMI 85th-95th centile).

Thirty healthy normal-weight children of matched age and sex were taken as controls.

Inclusion criteria: children aged 5 to 15 years with obesity or overweight, having a duration of more than 6 months, and on an unrestricted diet.

Exclusion criteria: Obese and overweight children with endocrine disorders, genetic syndromes, chronic systemic diseases (e.g., liver, renal, cardiac, chest, etc.), children on medication affecting glucose metabolism, such as glucocorticoids, valproate, psychotropic drugs, and those with acute illnesses causing inflammatory changes, were excluded

### Clinical assessment

All children included in the study underwent a comprehensive history and medical assessment, including anthropometric measurements.

For anthropometric measurements, children were dressed in light underwear and were either barefoot or wore socks. All body measurements were taken using standard equipment following the recommendations of the International Biological Program [9]. The average of three consecutive readings was used to determine each measurement. The Seca scale measured body weight to the nearest 0.1 kg, while the Harpenden stadiometer was used to measure height to the nearest 0.1 cm [10]. BMI was calculated as follows: BMI = weight/height^2 (kg/m^2). Children were classified as overweight if their BMI was above the 85th to 95th percentile, and obese if their BMI was at or above the 95th percentile [11]. Z-scores for height, weight, and BMI were also calculated. Values from Egyptian growth curves were displayed [12, 13]. Waist circumference (WC) was measured at the midaxillary line, halfway between the inferior margin of the ribs and the top of the hip bone [14].

Blood pressure was measured with the child seated, following the guidelines of the Seventh Report of the Joint National Committee on Prevention, Detection, Evaluation, and Treatment of High Blood Pressure [15].

### Pubertal assessment

Pubertal status was classified through direct examination using Tanner’s staging criteria (Tanner Stages I-V), based on secondary sexual characteristics (breast and pubic hair development in girls; genital and pubic hair development in boys). A single qualified investigator performed Tanner’s pubertal staging [16].

### Laboratory investigations

Venous blood samples were drawn from the antecubital vein while the subject was in the supine position. Samples were collected between 8:00 and 10:00 am after fasting for at least 8–10 h, then centrifuged at 3000 RPM for 15 min to measure fasting blood glucose (FBG), fasting insulin, HbA1c, and lipid profile. Triglycerides, HDL-cholesterol (HDL-c), and low-density lipoprotein cholesterol (LDL-c) were enzymatically measured using spectrophotometric methods (The Thermo Scientific Konelab 20 Clinical Chemistry Analyzer). Insulin levels were determined by microparticle enzyme immunoassay. HbA1c was measured with the Fluorescence Immunochromatographic Analyzing System (Finecare TM FIA Meter Plus).

Oral glucose tolerance test (OGTT): Fasting blood samples were collected to determine FBG. Subsequently, the child drank a glass of glucose solution (1.75 g/kg of dextrose, with a maximum of 75 g, dissolved in 250 ml of water). Blood glucose levels were measured at 0, 30, 60, 90, and 120 min during the OGTT, and all intermediate values were included in the analysis to evaluate glucose kinetics among study groups [17]. Plasma glucose concentrations were determined using the glucose-oxidase method.

### Definitions and calculations

- Fasting plasma glucose (FBG) levels of ≥ 100 mg/dL but < 126 mg/dL, or 2-h post-load glucose levels of < 140 mg/dL, are defined as impaired fasting glucose (IFG). Impaired glucose tolerance test (IGT) is diagnosed by a plasma 2 h postprandial glucose level ≥ 140 mg/dL but < 200 mg/dL, while T2DM is diagnosed when the 2-h postprandial plasma glucose level is ≥ 200 mg/dL. The normal glucose tolerance test is characterized by fasting plasma glucose levels of < 100 mg/dL and 2-h post-load plasma glucose concentrations of < 140 mg/dL [18].
- HOMA-IR: fasting plasma glucose (mg/dl) times fasting serum insulin (mU/l) divided by 405 [19]. HOMA-IR values between 0.5 and 1.4 are considered normal, ≥ 1.9 indicate early IR, and ≥ 2.9 indicate IR as in adults [20]. There is no universally accepted international guideline for pediatric HOMA-IR cut-offs, so we proposed age and puberty specific cut-offs rather than fixed cut-offs in children
- The TyG index was calculated using the following formula: Ln [fasting triglycerides (mg/dL) × fasting glucose (mg/dL)]/2 [21].

### Outcomes

The primary outcome of this study was to evaluate the potential of the TyG index in detecting GD, including IR, IFT, IGT, and T2DM in obese and overweight children. The secondary goal was to correlate the TyG index with HbA1C, FBG, 2-h PP glucose level, and HOMA-IR in these children.

### Statistical analysis

Power analysis revealed that a sample size of 30 children in each group was necessary to achieve a power of 80% with alpha = 0.05. Statistical analysis was done by SPSS v27 (IBM©, Chicago, IL, USA). The Shapiro-Wilks test was used to evaluate the normality of the data. Quantitative parametric data were presented as mean and standard deviation (SD) and were analyzed by one-way analysis of variance ANOVA (F) test with post hoc test (Tukey). Comparison between any two groups was performed utilizing the unpaired Student’s *t*-test. Qualitative variables were presented as frequency and percentage (%) and were analyzed utilizing the Chi-square test. Pearson correlation was done to estimate the degree of correlation between two quantitative variables. Sensitivity, specificity, and cut-off points of the TyG were examined using receiver-operating characteristic (ROC) curve analyses. P < 0.05 was accepted as the limit value of significance.

## Results

Group 1 included 30 children with obesity; 13 males and 17 females, with a mean age of 10.3 ± 2.83 years, and group 2 included 30 children with overweight; 17 males and 13 females, with a mean age of 10.7 ± 3.14 years. Age, sex, history of fracture and DM, height, SBP, and DBP were not significantly different among the three groups.

As expected, weight, weight -SDS, height- SDS, BMI, and BMI-SDS were significantly higher in the overweight and obese groups compared to the control group, with the highest values observed in group 1 (P < 0.05), since these parameters were used as defining criteria for group classification. Waist circumference was significantly greater in group 1 than in both group 2 and the control group (P < 0.001), with no significant difference between group 2 and the control group. Total cholesterol, HDL-c, LDL-c, and TG were all significantly higher in groups 1 and 2 compared with the control group (P < 0.05), with no significant difference between groups 1 and 2 regarding HDL-c and LDL-c. HbA1c, FBG, 2-h postprandial glucose, and TyG index were significantly higher in groups 1 and 2 than in the control group (P < 0.05), with the highest values in group 1. HOMA-IR was significantly higher in groups 1 and 2 than in the control group, with no significant difference between groups 1 and 2 (Table 1).

**Table 1:** Demographic, anthropometric, and laboratory data of the studied groups

Variables	Group 1	Group 2	Control group	*P* value
Age (Years)	10.3 ± 2.83	10.7 ± 3.14	9.9 ± 2.94	NS
Sex (male: female)	13:17	17:13	10:20	NS
History of fracture	2 (6.67%)	3 (10%)	0 (0%)	NS
History of DM	9 (30%)	5 (16.67%)	3 (10%)	NS
Weight (kg)	52.6 ± 6.82^†^	47.1 ± 11.56^†^	40.3 ± 9.25	< 0.001
Weight-SDS	3.5 ± 0.73^*†^	1 ± 0.61^†^	0.3 ± 0.97	< 0.001
Height (cm)	143 ± 8.37	144.7 ± 10.58	146 ± 8.36	NS
Height-SDS	2.6 ± 0.65^*†^	1.2 ± 0.57^†^	0.4 ± 1.18	< 0.001
BMI (kg/m^2^)	25.8 ± 3.26^*†^	22.3 ± 4.23^†^	18.8 ± 3.6	< 0.001
BMI-SDS	3.2 ± 0.72^*†^	2 ± 0.17^†^	0.1 ± 0.85	< 0.001
WC (cm)	83.4 ± 5.18^*†^	64.5 ± 6.58	63.5 ± 7.5	< 0.001
SBP (mmHg)	102.9 ± 13.3	103 ± 12.9	100.6 ± 13.1	NS
DBP (mmHg)	76 ± 5.7	75.1 ± 6.8	72.4 ± 6.7	NS
Total cholesterol (mg/dl)	197.5 ± 27.05^*†^	176.6 ± 23.14^†^	152.6 ± 9.97	< 0.001
HDL-c (mg/dl)	130.9 ± 26.67^†^	127.5 ± 8.07^†^	109.6 ± 7.38	< 0.001
LDL-c (mg/dl)	44 ± 5.88^†^	44.5 ± 5.62^†^	40.5 ± 2.87	< 0.001
TG (mg/dl)	78.9 ± 2.5^*†^	72.8 ± 3.0^†^	69.5 ± 1.0	< 0.001
HbA1C	5.9 ± 0.6^*†^	5.4 ± 0.4^†^	4.5 ± 0.3	< 0.001
FBG (mg/dl)	94.7 ± 19.5^*†^	81.1 ± 14.9^†^	70.8 ± 0.7	< 0.001
2-h postprandial glucose (mg/dL)	143.9 ± 25.7^*†^	114.6 ± 22.2^†^	108.1 ± 10.8	0.002
HOMA-IR	3.75 ± 0.70^†^	3.26 ± 0.89^†^	1.43 ± 0.68	< 0.001
TyG index	4.44 ± 0.10^*†^	4.32 ± 0.08^†^	4.24 ± 0.01	< 0.001

*NS* non-significant, *means *P* value is significant compared to group 2, ^†^means that *P* value is significant compared to the control group, *DM* diabetes mellitus, *significant as *P* value ≤ 0.05, *BMI* body mass index, *Weight-SDS* weight standard deviation score, *Height-SDS* height standard deviation score, *BMI-SDS* body mass index standard deviation score, *WC* waist circumference, *SBP* systolic blood pressure, *DBP* diastolic blood pressure, *HDL-c* high density lipoprotein cholesterol, *LDL-c* low density lipoprotein cholesterol, *HbA1C* Glycated haemoglobin, *HOMA-IR* Homeostatic Model Assessment for Insulin Resistance, *TyG* triglyceride glucose

Fasting blood glucose as well as blood glucose levels at 0.5 h, 1 h, 1.5 h, and 2 h were significantly higher in obese/overweight children compared to normal weight children (Table 2).

**Table 2:** Blood glucose levels in the oral glucose tolerance test in the studied groups

Variables	Overweight/obese group (*N* = 60)	Normal weight group (*N* = 30)	*P* value
Fasting blood glucose (mg/dl)	91.78 ± 20.54	70.80 ± 0.76	0.001
0.5-h postprandial glucose (mg/dl)	134.55 ± 30.61	117.83 ± 8.49	0.026
1-h postprandial glucose (mg/dl)	149.95 ± 50.16	124.53 ± 7.49	0.037
1.5-h postprandial glucose (mg/dl)	144.98 ± 51	112.4 ± 8.33	0.001
2-h postprandial glucose (mg/dl)	138.58 ± 53.63	108.13 ± 10.83	0.003

HbA1c and HOMA-IR were significantly higher in overweight/obese children compared with normal weight children. However, FBG, 2-h postprandial glucose, the TyG index, and triglyceride levels were insignificantly different between the two groups (Table 3).

**Table 3:** HbA1c, fasting blood glucose, 2-h postprandial glucose, HOMA-IR, TyG index, and triglycerides in overweight/obese children vs normal weight children

Variable	Overweight/obese children (*n* = 20)	Normal weight children (*n* = 13)	*P*
HbA1c	5.26 ± 0.31	4.50 ± 0.26	< 0.001
Fasting blood glucose (mg/dl)	73.6 ± 8.55	70.85 ± 0.80	0.676
2-h postprandial glucose (mg/dl)	117.05 ± 24.89	106 ± 10.36	0.169
HOMA-IR	2.79 ± 0.54	1.72 ± 0.63	< 0.001
The TyG index	4.28 ± 0.10	4.24 ± 0.01	0.928
Triglyceride (mg/dl)	72.20 ± 8.37	69.62 ± 1.04	0.870

*HbA1C* glycated haemoglobin, *HOMA-IR* Homeostatic Model Assessment for Insulin Resistance, *TyG* triglyceride glucose

HbA1c, HOMA-IR, FBG, 2-h postprandial glucose, the TyG index, and triglyceride levels were significantly higher in overweight/obese adolescents compared with normal weight adolescents (Table 4).

**Table 4:** HbA1c, fasting blood glucose, 2-h postprandial glucose, HOMA-IR, TyG index, and triglycerides in overweight/obese adolescents vs normal weight adolescents

Variables	Overweight/obese adolescents (*n* = 40)	Normal weight adolescents (*n* = 17)	*P*
HbA1c	5.90 ± 0.60	4.62 ± 0.38	< 0.001^*^
Fasting blood glucose (mg/dl)	95.05 ± 18.11	70.76 ± 0.75	< 0.001^*^
2-h postprandial glucose (mg/dl)	135.45 ± 48.47	109.76 ± 11.21	0.027^*^
HOMA-IR	3.86 ± 0.71	1.21 ± 0.65	< 0.001^*^
The TyG index	4.43 ± 0.15	4.24 ± 0.01	< 0.001^*^
Triglyceride (mg/dl)	77.68 ± 11.63	69.41 ± 1.06	< 0.001^*^

*HbA1C* glycated haemoglobin, *HOMA-IR* Homeostatic Model Assessment for Insulin Resistance, *TyG* triglyceride glucose

The prevalence of T2DM, IGT, and IFG was 26.6%, 16.7%, and 36.7% respectively in the obese group, and 0%, 10%, and 23.3% respectively in the overweight group (Table 5).

**Table 5:** Prevalence of T2DM, IGT, and IFG in the studied groups

Variables	Frequency of the studied groups	
	Group I	Group II
	Obese group (*n* = 30)	Overweight group (*n* = 30)
T2DM	8 (26.6%)	0 (0%)
IGT	5 (16.7%)	3 (10%)
IFG	11 (36.7%)	7 (23.3%)

*T2DM* type 2 diabetes mellitus, *IGT* impaired glucose tolerance, *IFG* impaired fasting glucose

There was a strong positive correlation between TyG index and (HbA1c, fasting blood glucose, HOMA IR, and 2-h postprandial glucose) (Table 6).

**Table 6:** Correlation between TyG index and HOMA-IR, HbA1c, fasting blood glucose, 2-h postprandial blood glucose

Variables	TyG index	
	*R*	*P*
HBAIC (%)	0.703	< 0.001
Fasting blood glucose	0.875	< 0.001
HOMA-IR	0.675	< 0.001
2-h postprandial glucose	0.404	< 0.001

*HbA1C* glycated hemoglobin, *HOMA-IR* Homeostatic Model Assessment for Insulin Resistance, *TyG* triglyceride glucose

**Fig. 1:**
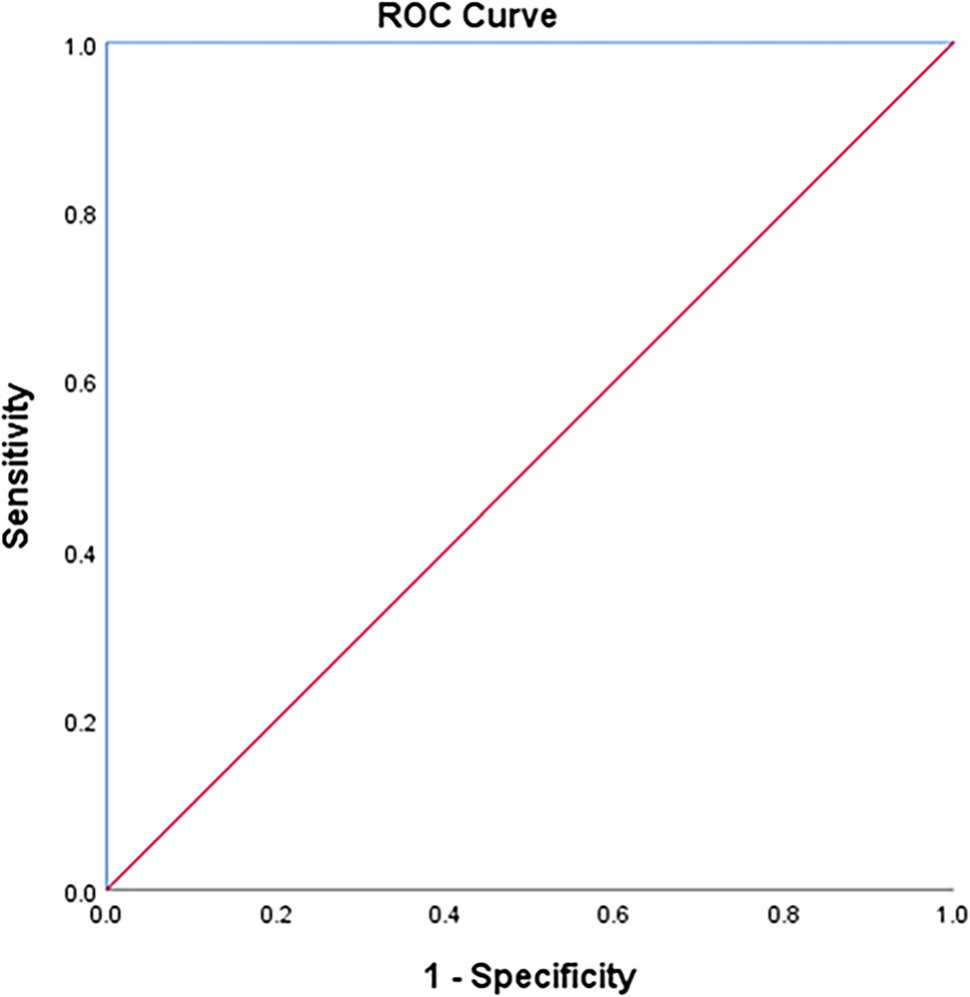
ROC curve of the TyG index to predict T2DM at a cut-off ≥ 4.57 with 100% sensitivity and 95.3% specificity

**Fig. 2:**
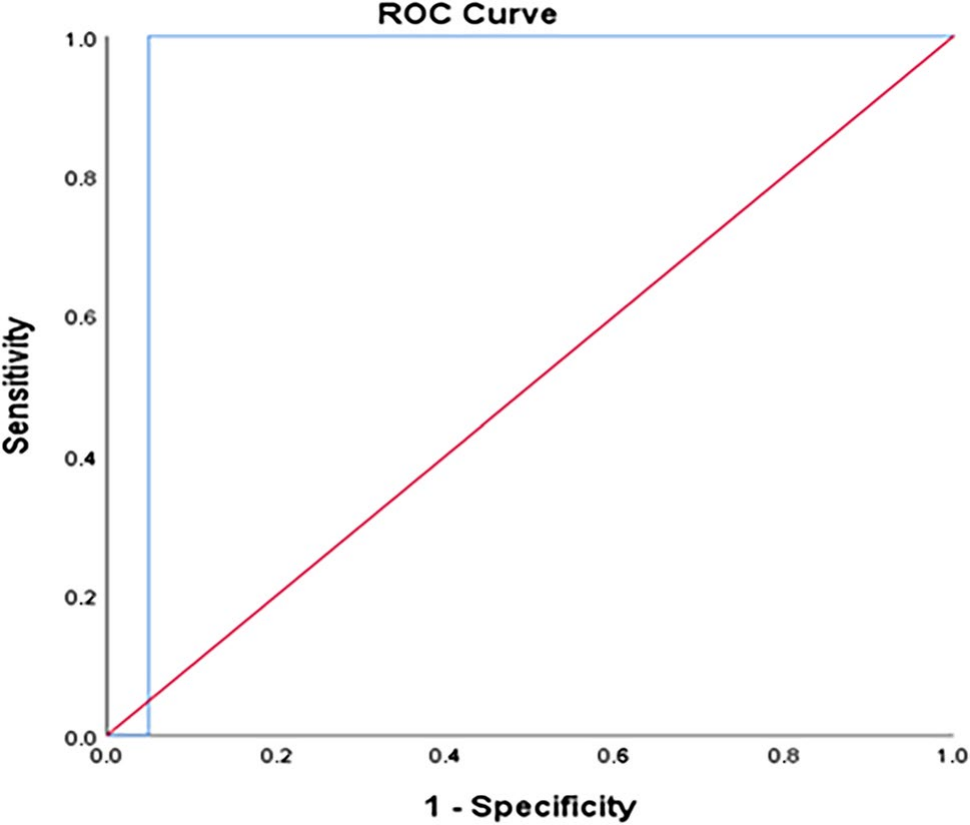
ROC curve of the TyG index to predict IGT at a cut-off ≥ 4.52 with 100% sensitivity and 95.1% specificity

**Fig. 3:**
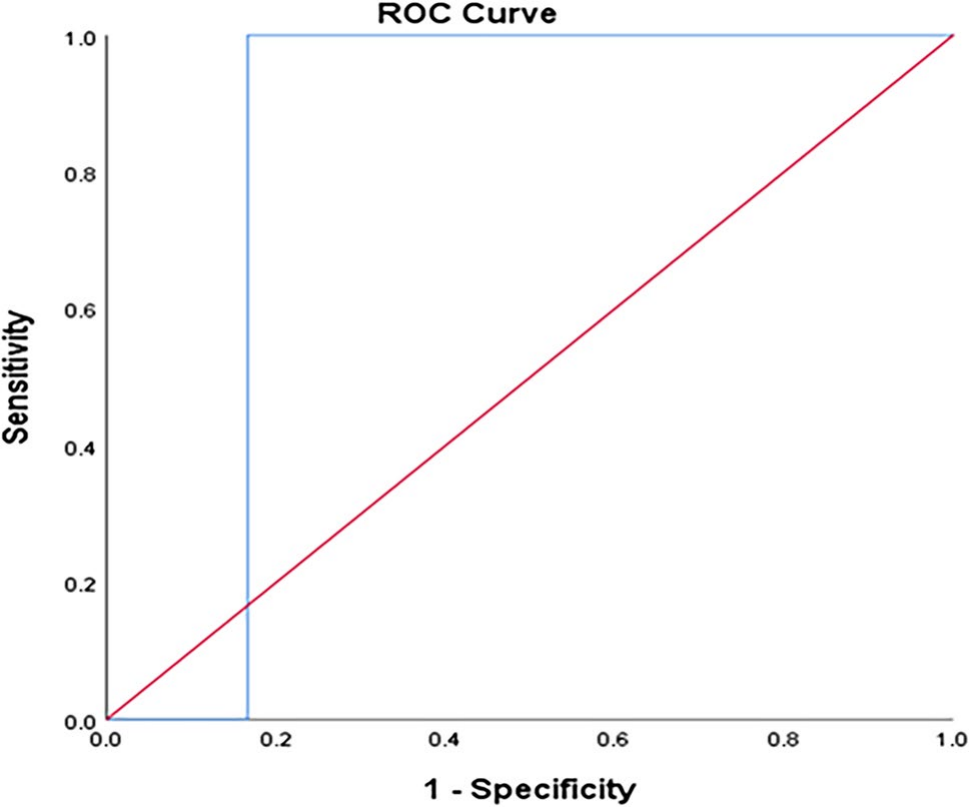
ROC curve of the TyG index to predict IFT at a cut-off ≥ 4.43 with 88.9% sensitivity and 88.3% specificity

**Fig. 4:**
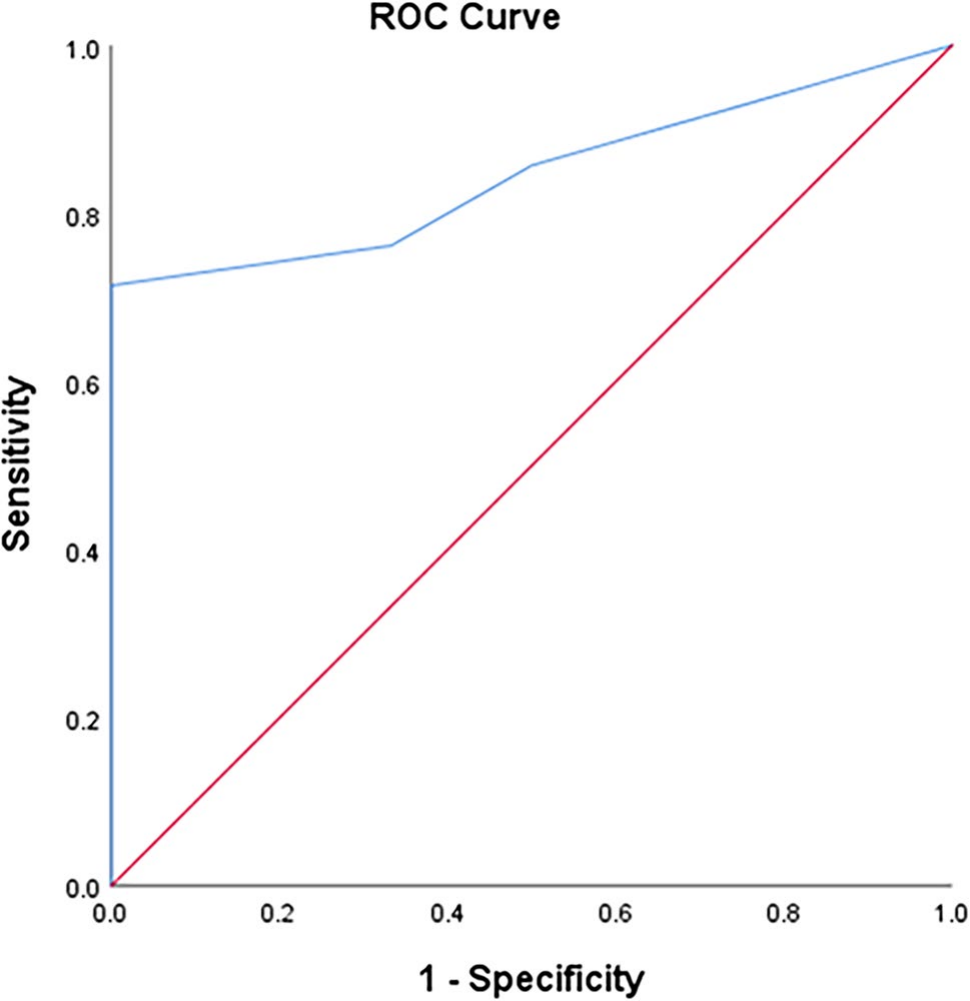
ROC curve of the TyG index to predict insulin resistance at a cut-off ≥ 4.34 with 71.4% sensitivity and 100% specificity

The TyG index can significantly predict T2DM at a cut-off ≥ 4.57 with 100% sensitivity and 95.3% specificity (Fig. 1). The TyG index can significantly predict IGT at a cut-off ≥ 4.52 with 100% sensitivity and 95.1% specificity (Fig. 2). The TyG index can significantly predict IFT at a cut-off ≥ 4.43 with 88.9% sensitivity and 88.3% specificity (Fig. 3). The TyG index can significantly predict insulin resistance at a cut-off ≥ 4.34 with 71.4% sensitivity and 100% specificity (Fig. 4).

## Discussion

The prevalence of childhood obesity has risen in recent decades due to changes in diet and lifestyle [22]. Obese children exhibit metabolic abnormalities such as glucose disorders. While several clinical and biochemical markers for insulin resistance and glucose disorders exist, many require expensive and less reproducible methods. It’s essential to expand the knowledge of more accessible markers that would allow healthcare providers to identify obese patients with higher metabolic risks and glucose disorders for specialized care [23]. The TyG index has been studied in adults, showing modest correlations with the HOMA index; it appears to be a promising predictor of insulin resistance and glucose disorders in the pediatric population [24].

In the current study, increased serum lipid profile was observed in both obese and overweight children compared to the controls. This was in agreement with previous studies [25–28].

Regarding our results, HbA1c, FBG, HOMA-IR, and the TyG index were significantly higher in Group 1 (obese) compared to Group 2 (overweight) and significantly higher in both groups compared to the control group. The 2-h postprandial glucose levels were significantly higher in Group 1 than in Group 2 and the control group. In agreement with our results, García et al. [27] found a statistically significant difference between the obese-overweight group and the normal-weight group as regards HOMA-IR and the TyG index.

Our findings can be explained by the fact that excessive accumulation of body fat contributes to the development of T2DM, and the risk of T2DM increases proportionally with the increase in BMI. Consequently, the worldwide increase in the prevalence of obesity has led to a concomitant increase in the prevalence of T2DM. The underlying cellular and physiological mechanisms linking obesity to T2DM are complex and involve adiposity-induced alterations in β-cell function, adipose tissue biology, and multi-organ insulin resistance. Importantly, these pathological changes can often be improved- and in some cases reversed- through adequate weight loss [29].

Currently, there are no universally accepted international guidelines defining HOMA-IR cut-off values for insulin resistance in pediatric populations [20], there is emerging evidence in children and adolescents for alternative age, sex or puberty-adjusted values. There are several pediatric relevant studies that propose alternative thresholds. For example, in an Egyptian cohort of obese children, a HOMA-IR of 3.4 (90th percentile) was used to define insulin resistance [30]. In another large adolescent cohort (ERICA, Brazil), optimal HOMA-IR thresholds associated with metabolic syndrome were 2.80 overall, 2.32 in girls and 2.87 in boys [31]. Another study proposed puberty- and gender-specific HOMA-IR cut-offs (e.g. ~ 2.67 in prepubertal boys, ~ 5.22 in pubertal boys, ~ 2.22 in prepubertal girls and ~ 3.82 in pubertal girls) [32]. Therefore, we advocated for age, sex and puberty specific cutoff points that vary with age and puberty rather than fixed values. These findings highlight the importance of future local studies to define validated, population-specific pediatric cut-offs.

In our study, HbA1c, FBG, HOMA-IR, the TyG index, and triglyceride were significantly higher in overweight/obese adolescents compared with normal weight adolescents. Moreover, HbA1c and HOMA-IR were significantly higher in overweight/obese children compared with normal weight children but FBG, 2-h post prandial glucose, the TyG index, and triglycerides were insignificantly different between two groups. These findings can be explained by the fact that there is a physiological insulin resistance associated with puberty. During adolescence, increased secretion of growth hormone, insulin-like growth factor-1 (IGF-1), and sex steroids induces a transient decline in insulin sensitivity which typically reaches its nadir in mid-puberty and recovers upon completion of pubertal maturation. Excess adiposity further aggravates this physiological insulin resistance, leading to more pronounced metabolic disturbances in obese and overweight adolescents. In contrast, prepubertal children, who have not yet undergone these hormonal changes, maintain relatively stable insulin sensitivity, which may explain the absence of significant metabolic differences between weight groups in this age range [33–36]

In our study, a strong positive correlation was observed between the TyG index and several metabolic parameters, including HBA1C, FBG, HOMA IR, and 2-h postprandial glucose. These findings are consistent with the results of Sánchez et al. [37] who reported a significant positive correlation between the TyG index and other measures of IR, such as blood insulin levels and the HOMA-IR. Similarly, studies conducted on overweight and obese children demonstrated that the TyG index is positively correlated with fasting glucose, HOMA-IR, and HbA1C [38–40].

The TyG index has emerged as a reliable surrogate marker for IR, with its utility supported by multiple studies across diverse populations. Insulin resistance plays a pivotal role in the progression from prediabetes and T2DM. Prediabetes, which includes impaired fasting glucose (IFG) and impaired glucose tolerance (IGT), is characterized by varying degrees of insulin sensitivity and secretion, alongside abnormalities in hepatic glucose production and incretin hormone activity. These alterations collectively contribute to the pathogenesis of diabetes [41].

We found that the TyG index can significantly predict IFT at a cut-off value of ≥ 4.43, with 88.9% sensitivity and 88.3% specificity. IGT was best predicted at a cut-off value of ≥ 4.52, yielding 100% sensitivity and 95.1% specificity, while T2DM was predicted at a cut-off ≥ 4.57, with 100% sensitivity and 95.3% specificity. In agreement with our results, Simental et al. [42] evaluated the diagnostic utility of the TyG index for identifying glucose disorders in apparently healthy children and adolescents. Their study reported optimal TyG cut-off values of 4.51 for IFG (sensitivity 59.8%, specificity 59.8%), 4.55 for IGT (sensitivity 63.0%, specificity 64.3%), and 4.63 for T2DM (sensitivity 75.0%, specificity 74.6%) in girls. For boys, the respective cutoff values were 4.52 for IFG (sensitivity 62.8%, specificity 64.2%), 4.54 for IGT (sensitivity 71.8%, specificity 65.1%), and 4.82 for T2DM (sensitivity 91.0%, specificity 90.6%). Moreover, Wang et al. [43] evaluated the association between the TyG index and incident T2DM in a prospective Chinese cohort, and they found that T2DM increased with a TyG index > 8.51 by 38%^.^ These results suggest that the TyG index may provide higher diagnostic accuracy in our cohort compared to previously reported cut-offs.

We also found that the TyG index can significantly predict IR at a cut-off value ≥ 4.34, with 71.4% sensitivity and 100% specificity. Insulin resistance is one of the earliest metabolic complications in obese children. In our study, most patients exhibited insulin resistance, and this may be linked to abdominal obesity. Recognizing these initial abnormalities using the TyG index in primary care could facilitate early specialized interventions, potentially preventing progression to metabolic syndrome. In agreement with our results, Locateli et al. [6] found that the TyG index cut-off value > 4.44 can predict IR, with a sensitivity of 75.7% and a specificity of 67.4%. Moreover, Sánchez et al. [37] reported the best cut-off point for the TyG index for predicting IR was 4.21 in prepubertal children (sensitivity 84%, specificity 100%) and 4.33 in adolescents (sensitivity 89%, specificity 69%).

The causes of differences in sensitivity and specificity for the TyG between our study and previous studies are several methodological and population-related factors. First, our relatively small sample size and case mix (proportion of overweight vs. obese and the distribution of glucose abnormalities) differ from previous published cohorts. Second, differences in pubertal status and age distribution that affect insulin dynamics. Third, variation in cut-off selection methods and laboratory assays (different assays for insulin, glucose measurement techniques, fasting duration, and pre-analytical handling) can systematically change TyG values. Finally, ethnic and genetic differences may also play a role.

The strengths of our study included the use of the TyG index to detect different glucose disorders in overweight and obese children and adolescents, not just IR, as in previous studies.

Limitations of the study: The small sample size and being a single-center study were the main limitations of the study.

## Conclusion

The TyG index is a promising marker for predicting different glucose disorders in obese children and adolescents.

## Data Availability

All the data of the study are available from the corresponding author on reasonable request.

## Abbreviations

ANOVA: Analysis of variance
BMI: Body mass index
BP: Blood pressure
DBP: Diastolic blood pressure
FBG: Fasting blood glucose
GD: Glucose disorders
HDL-c: High-density lipoprotein cholesterol
HOMA-IR: Homeostatic Model Assessment for Insulin Resistance
IFG: Impaired fasting glucose
IGT: Impaired glucose tolerance
IR: Insulin resistance
LDL-c: Low-density lipoprotein cholesterol
ROC: Receiver operating characteristics
SBP: Systolic blood pressure
SD: Standard deviation
TyG: Triglyceride glucose
T2DM: Type 2 diabetes mellitus
